# Benefits of intravenous iron supplementation in heart failure

**DOI:** 10.21542/gcsp.2024.10

**Published:** 2024-03-03

**Authors:** Susy Kotit

**Affiliations:** Aswan Heart Centre, Aswan, Egypt

## Abstract

**Introduction:** Iron deficiency (ID) is one of the most frequent comorbidities in patients with heart failure (HF) and is estimated to be present in up to 80% of acute patients regardless of their ejection fraction. Randomized controlled trials have shown that supplementary intravenous iron results in improved clinical outcomes; however, the current understanding of the effects of intravenous iron on morbidity and mortality remains limited.

**Study and results:** The meta-analysis pooled individual participant data from three randomized placebo-controlled trials of ferric carboxymaltose (FCM) in adult patients (*n* = 4,501) with heart failure and iron deficiency (CONFIRM-HF, AFFIRM-AHF, and HEART-FID). FCM therapy significantly reduced the co-primary composite endpoint of total cardiovascular hospitalizations and cardiovascular death, with a rate ratio (RR 0.86; 95% CI 0.75 to 0.98; *p* = 0.029). FCM therapy was associated with a 17% relative rate reduction in total cardiovascular hospitalizations (RR 0.83; 95% CI 0.73 to 0.96; *p* = 0.009) and a 16% relative rate reduction in total heart failure hospitalizations (RR 0.84; 95% CI 0.71 to 0.98; *p* = 0.025).

**Lessons learned:** The meta-analysis shows that in iron-deficient patients with heart failure and reduced or mildly reduced left ventricular ejection fraction, intravenous ferric carboxymaltose (FCM) is associated with a reduced risk of total cardiovascular hospitalization and cardiovascular mortality. These findings indicate that intravenous FCM should be considered in iron-deficient patients with heart failure and reduced or mildly reduced ejection fractions.

## Introduction

Iron deficiency (ID) is one of the most frequent comorbidities in patients with heart failure (HF)^1–6^ and is estimated to be present in up to 80% of patients^2,4,7–21^, regardless of their ejection fraction^6,11^, and is a strong independent predictor of HF outcomes^2,4,22–25^.

In patients with HF, ID is associated with reduced quality of life^1–6,22^, exercise capacity^1–6^, peak VO_2_^24^, and survival^6^, and an increased risk of hospitalization^6^, independent of demographics and clinical variables, including anaemia^2,4,23–26^.

Iron is the most important essential trace element in the body, as it maintains the oxygen-carrying capacity of the blood through erythropoiesis and is independently crucial for oxygen uptake, transport, storage, and metabolism, as well as cellular immune responses^27,28^. In addition, iron serves as a fundamental component of hemoglobin, myoglobin, and diverse enzymes involved in cellular respiration, nitric oxide generation, oxidative phosphorylation, the citric acid cycle, oxygen radical production, and other vital cellular and body functions^29^.

Metabolic active cells with high energy demands, such as skeletal muscle cells and myocytes, depend on iron for their structural integrity and function^10,27,30–32^. At the cellular level, ID decreases enzymatic activity of both the Krebs cycle and the respiratory chain in the mitochondria, leading to disturbance in the energetic metabolism of cells^33^. ID can therefore decrease oxygen storage in myoglobin and reduce tissue oxidative capacity, causing structural and functional change in the myocardium, leading to mitochondrial and myocardial dysfunction^34,35^, and adverse remodelling.

**Figure 1.**
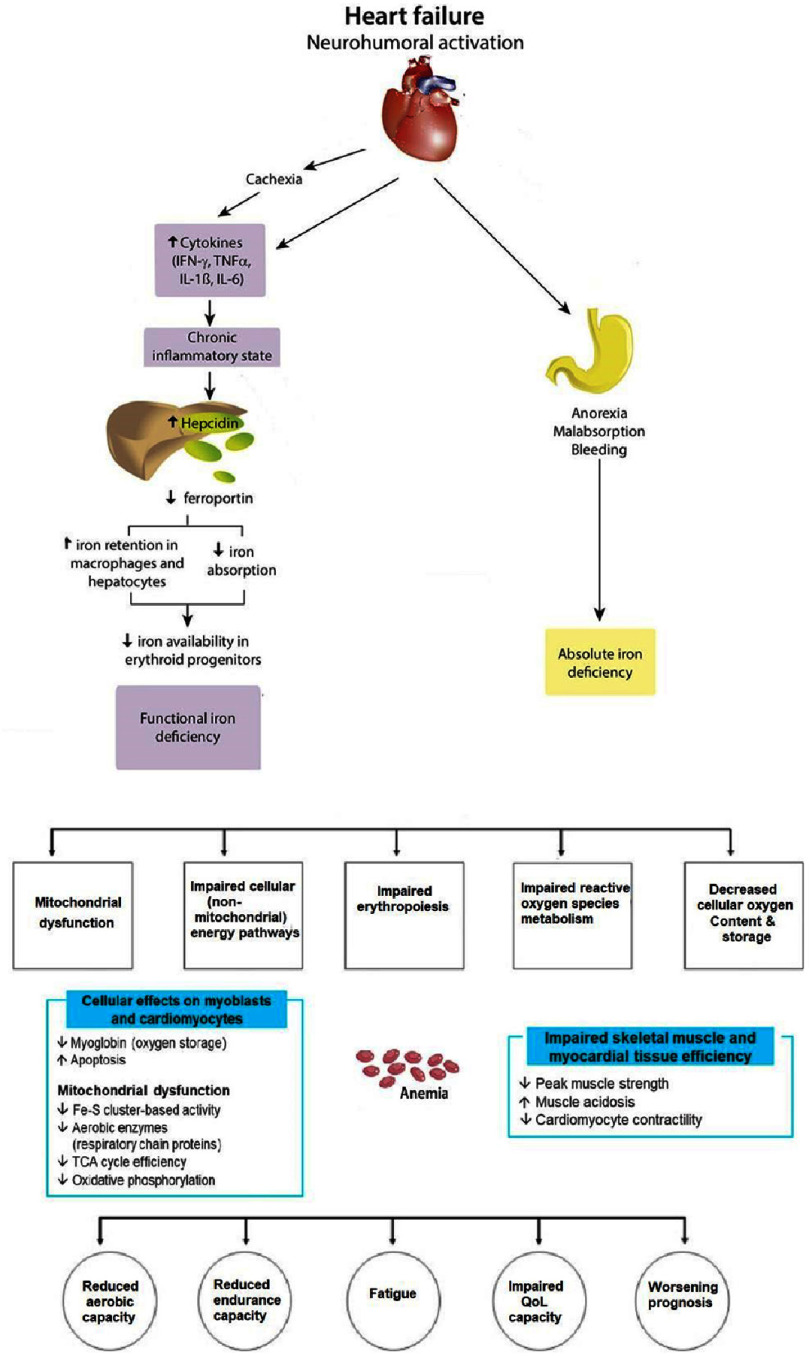
Iron deficiency in heart failure^32,71–76^.

Furthermore, reduced oxygen delivery to metabolizing tissues triggers proinflammatory cytokine activation^36–38^, as well as hemodynamic, neurohormonal, and renal alterations^38^, leading to increased myocardial workload, adverse myocardial remodelling, left ventricle hypertrophy^39,40^, progressive fibrosis^35,41–47^, reduced exercise capacity^35,48–50^ and decline in prognosis (Figure 1).

Moreover, patients with HF and ID frequently have several comorbidities, including chronic kidney disease, cardiac cachexia-associated poor nutritional status, and low albumin levels^51–53^, all of which have a significant impact on outcomes.

The 2021 European Society of Cardiology (ESC) guidelines on HF acknowledge the importance of iron deficiency and provide specific recommendations for the diagnosis and treatment of ID^54^. However, iron deficiency remains under-recognized and undertreated in clinical practice^18,55–58^, partially owing to a lack of practical guidance for clinicians.

Importantly, oral iron administration, initially the first route used for iron repletion, has not demonstrated any benefit in patients with HF and reduced ejection fraction, as it did not affect peak VO_2_ (the primary endpoint of the study) or increase serum ferritin levels^59^.

In contrast, randomized controlled trials have shown that in patients with heart failure and reduced ejection fraction, supplementary intravenous iron results in improvements in symptoms, functional capacity, peak oxygen consumption^60^, quality of life^60–66^, and decreased risk of first hospitalization for worsening HF^66,67^. Consequently, correction of iron deficiency in patients with HF and reduced ejection fraction (EF) with intravenous ferric carboxymaltose is now recommended to improve clinical outcomes^54^. Although the clinical and prognostic significance of ID in HF is now widely acknowledged^12,68,69^, our current understanding of the effects of intravenous iron on morbidity and mortality remains limited^70^.

## The meta-analysis

This meta-analysis pooled individual participant data from three randomized, placebo-controlled trials of intravenous ferric carboxymaltose (FCM) in adult patients with heart failure and iron deficiency with at least 52 weeks of follow-up (CONFIRM-HF^61^, AFFIRM-AHF^66^, and HEART-FID^77^) to evaluate the effects of FCM therapy on hospitalization and mortality in iron-deficient patients with heart failure and reduced or mildly reduced left ventricular ejection fraction (LVEF).

The analysis had two primary efficacy endpoints that were examined through 52 weeks of follow-up: (1) composite of total cardiovascular hospitalizations and cardiovascular death and (2) composite of total heart failure hospitalizations and cardiovascular death. The prospectively recorded clinical outcomes included first and recurrent HF and CV hospitalizations, CV death, and all-cause mortality.

The CONFIRM-HF trial included ambulatory HF patients in New York Heart Association (NYHA) class II–III, with left ventricular ejection fraction (LVEF) ≤45% and elevated natriuretic peptide levels^61^. The AFFIRM-AHF trial recruited patients hospitalized for acute HF with LVEF <50%^66^ and the HEART-FID trial enrolled patients with HF and LVEF ≤40% who had recent (within 12 months) hospitalization for HF and/or elevated natriuretic peptide levels^77^.

Iron deficiency was reported using the same definition across all three trials: ferritin <100 ng/mL or ferritin 100-300 ng/mL with a transfer <20%).

## Results

**Figure 2.**
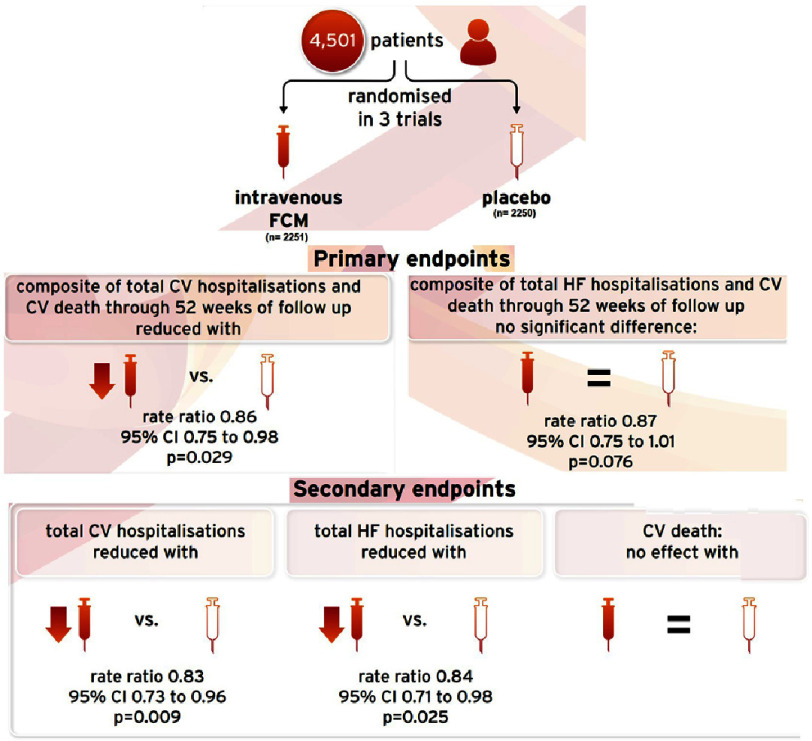
Meta-analysis design and results^78^.

Over the three trials, a total of 4,501 patients with heart failure, reduced left ventricular ejection fraction, and iron deficiency were randomly assigned to FCM (*n* = 2,251) or placebo (*n* = 2,250) (Figure 2). The mean age of the total population was 69.2 years, 63% were men, and the mean left ventricular ejection fraction was 31.6%.

The meta-analysis showed that compared with placebo, FCM therapy significantly reduced the co-primary composite endpoint of total cardiovascular hospitalization and cardiovascular death, with a rate ratio (RR) of 0.86 (95% confidence interval [CI] 0.75 to 0.98; *p* = 0.029). Although statistically non-significant, there was a trend towards reduction of the co-primary composite endpoint of total heart failure hospitalizations and cardiovascular death (RR, 0.87; 95% CI 0.75 to 1.01; *p* = 0.076).

FCM therapy was associated with a 17% relative rate reduction in total cardiovascular hospitalizations (RR 0.83; 95% CI 0.73 to 0.96; *p* = 0.009) and a 16% relative rate reduction in total heart failure hospitalizations (RR 0.84; 95% CI 0.71 to 0.98; *p* = 0.025).

FCM therapy reduced the time to first CV death or HF hospitalization by 12% (HR, 0.88; 95% CI [0.78–0.99]; *P* = 0.039) and the time to first CV death or CV hospitalization by 11% (HR 0.89; 95% CI [0.80–0.99]; *P* = 0.033).

Subgroup analyses showed that patients in the lowest transferrin saturation (TSAT) tertile (<15%) derived greater benefits from FCM for CV death (interaction *p* = 0.035) and the composite endpoint of total cardiovascular hospitalization or cardiovascular death (interaction *p* = 0.019) than those with higher baseline TSAT.

Importantly, FCM treatment appeared to be safe and well-tolerated.

## Discussion

This study represents the largest pooled meta-analysis using individual participant data to examine the effects of FCM therapy on hospitalization and mortality in iron-deficient patients with HF and reduced or mildly reduced LVEF.

The analysis showed that in iron-deficient patients with heart failure and reduced or mildly reduced LVEF, intravenous ferric carboxymaltose (FCM) was associated with a reduced risk of the composite outcome of total cardiovascular hospitalization and cardiovascular death through 52 weeks compared with placebo, with a statistically non-significant trend towards reduction of the rate of composite of CV death and total HF hospitalizations. Overall, the treatment appeared to be safe and well tolerated.

There was no evidence for the heterogeneity of treatment effects by sex, age, and baseline serum ferritin, concluding that FCM exerts favorable effects on clinical outcomes across subgroups^23,79,80^.

Importantly, patients with ischemic HF etiology tended to demonstrate greater benefits of FCM therapy regarding the reduction in HF hospitalization and CV death, indicating potential heterogeneity by HF etiology. However, this finding requires further investigation in prospectively designed studies with a robust definition of the underlying HF etiology.

Additionally, there was a difference in the effect of FCM on CV mortality among subgroups based on baseline TSAT, with statistically significant reductions in all-cause and CV mortality in patients with HF and the lowest TSAT values (<15%) and less favorable effects in patients with TSAT of 24% or greater.

According to the current understanding of ID in HF, intravenous iron therapy is often prescribed based on serum ferritin and TSAT levels. However, recent evidence suggests that these markers may not accurately reflect the depletion of iron in bone marrow or the iron status of peripheral target tissues, such as the myocardium or skeletal muscles^49,81–83^. Therefore, it is proposed that the current definition of ID in HF should be re-evaluated as the main indication for intravenous iron therapy.

Furthermore, a higher 6-month cumulative dose of ferric carboxymaltose as a result of re-dosing may be associated with a slightly greater treatment effect; however, additional research to identify eligibility criteria for an optimal re-dosing strategy is warranted.

In conclusion, this large meta-analysis provides further evidence that treatment with intravenous FCM significantly reduces recurrent HF and CV hospitalizations, with no new safety concerns. Importantly, the current study supports continued research to identify patients who are most likely to benefit from FCM treatment and the development of eligibility criteria for an optimal administration strategy.

## Lessons learned

The meta-analysis shows that in iron-deficient patients with heart failure and reduced or mildly reduced left ventricular ejection fraction, intravenous ferric carboxymaltose (FCM) is associated with a reduced risk of the composite outcome of total cardiovascular hospitalization and cardiovascular mortality over 52 weeks compared with placebo.

These findings indicate that intravenous FCM should be considered in iron-deficient patients with heart failure and reduced or mildly reduced ejection fraction to reduce the risk of hospitalization and adverse cardiovascular events.

Importantly, challenging the current definition of ID based on serum ferritin and TSAT levels as the main indication for intravenous iron therapy in patients with HF is warranted.

## References

[1] Jankowska EA (2010). Iron deficiency: an ominous sign in patients with systolic chronic heart failure. Eur Heart J.

[2] Klip IT (2013). Iron deficiency in chronic heart failure: an international pooled analysis. Am Heart J.

[3] Núñez J (2016). Iron deficiency and risk of early readmission following a hospitalization for acute heart failure. Eur J Heart Fail.

[4] Okonko DO, Mandal AKJ, Missouris CG, Poole-Wilson PA (2011). Disordered iron homeostasis in chronic heart failure: prevalence, predictors, and relation to anemia, exercise capacity, and survival. J Am Coll Cardiol.

[5] Alcaide-Aldeano A (2020). Iron deficiency: impact on functional capacity and quality of life in heart failure with preserved ejection fraction. J Clin Med.

[6] Martens P, Nijst P, Verbrugge FH, Smeets K, Dupont M, Mullens W (2018). Impact of iron deficiency on exercise capacity and outcome in heart failure with reduced, mid-range and preserved ejection fraction. Acta Cardiol.

[7] K. D. I. G. O. (KDIGO) A. W. Group (2012). KDIGO clinical practice guideline for anemia in chronic kidney disease. Kidney Int Suppl.

[8] Duarte JH (2014). Long-term iron therapy is beneficial in patients with HF. Nat Rev Cardiol.

[9] Anand IS, Gupta P (2018). Anemia and iron deficiency in heart failure: current concepts and emerging therapies. Circulation.

[10] Jankowska EA, von Haehling S, Anker SD, Macdougall IC, Ponikowski P (2013). Iron deficiency and heart failure: diagnostic dilemmas and therapeutic perspectives. Eur Heart J.

[11] Yeo TJ (2014). Iron deficiency in a multi-ethnic Asian population with and without heart failure: prevalence, clinical correlates, functional significance and prognosis. Eur J Heart Fail.

[12] von Haehling S, Jankowska EA, van Veldhuisen DJ, Ponikowski P, Anker SD (2015). Iron deficiency and cardiovascular disease. Nat Rev Cardiol.

[13] Nanas JN (2006). Etiology of anemia in patients with advanced heart failure. J Am Coll Cardiol.

[14] Parikh A, Natarajan S, Lipsitz SR, Katz SD (2011). Iron deficiency in community-dwelling US adults with self-reported heart failure in the National Health and Nutrition Examination Survey III: prevalence and associations with anemia and inflammation. Circ Heart Fail.

[15] von Haehling S (2017). Prevalence and clinical impact of iron deficiency and anaemia among outpatients with chronic heart failure: The PrEP Registry. Clin Res Cardiol.

[16] Cohen-Solal A (2022). Iron deficiency in heart failure patients: the French CARENFER prospective study. ESC Hear Fail.

[17] M. F. L. Rocha BML, Cunha GJL (2018). The burden of iron deficiency in heart failure: therapeutic approach. J Am Coll Cardiol.

[18] Cohen-Solal A (2014). High prevalence of iron deficiency in patients with acute decompensated heart failure. Eur J Heart Fail.

[19] Van Aelst LNL (2017). Iron status and inflammatory biomarkers in patients with acutely decompensated heart failure: early in-hospital phase and 30-day followup. European Journal of Heart Failure.

[20] Cappellini MD (2017). Iron deficiency across chronic inflammatory conditions: International expert opinion on definition, diagnosis, and management. Am J Hematol.

[21] Wexler D (2004). Prevalence of anemia in patients admitted to hospital with a primary diagnosis of congestive heart failure. Int J Cardiol.

[22] Comín-Colet J (2015). A cost-effectiveness analysis of ferric carboxymaltose in patients with iron deficiency and chronic heart failure in Spain. Rev Esp Cardiol (Engl Ed).

[23] Enjuanes C (2014). Iron deficiency and health-related quality of life in chronic heart failure: results from a multicenter European study. Int J Cardiol.

[24] Jankowska EA (2011). Iron deficiency predicts impaired exercise capacity in patients with systolic chronic heart failure. J Card Fail.

[25] van Veldhuisen DJ, Anker SD, Ponikowski P, Macdougall IC (2011). Anemia and iron deficiency in heart failure: mechanisms and therapeutic approaches. Nat Rev Cardiol.

[26] Comín-Colet J (2013). Iron deficiency is a key determinant of health-related quality of life in patients with chronic heart failure regardless of anaemia status. Eur J Heart Fail.

[27] Cairo G, Bernuzzi F, Recalcati S (2006). A precious metal: Iron, an essential nutrient for all cells. Genes Nutr.

[28] Haas JD, 4th Brownlie T (2001). Iron deficiency and reduced work capacity: a critical review of the research to determine a causal relationship. J Nutr.

[29] Dunn LL, Suryo Rahmanto Y, Richardson DR (2007). Iron uptake and metabolism in the new millennium. Trends Cell Biol.

[30] Melenovsky V (2017). Myocardial iron content and mitochondrial function in human heart failure: a direct tissue analysis. Eur J Heart Fail.

[31] Jankowska EA, Ponikowski P (2010). Molecular changes in myocardium in the course of anemia or iron deficiency. Heart Fail Clin.

[32] Stugiewicz M, Tkaczyszyn M, Kasztura M, Banasiak W, Ponikowski P, Jankowska EA (2016). The influence of iron deficiency on the functioning of skeletal muscles: experimental evidence and clinical implications. Eur J Heart Fail.

[33] Oexle H, Gnaiger E, Weiss G (1999). Iron-dependent changes in cellular energy metabolism: influence on citric acid cycle and oxidative phosphorylation. Biochim Biophys Acta.

[34] Brownlie IV T, Utermohlen V, Hinton PS, Haas JD (2004). Tissue iron deficiency without anemia impairs adaptation in endurance capacity after aerobic training in previously untrained women. Am J Clin Nutr.

[35] Dong F, Zhang X, Culver B, Chew Jr HG, Kelley RO, Ren J (2005). Dietary iron deficiency induces ventricular dilation, mitochondrial ultrastructural aberrations and cytochrome c release: involvement of nitric oxide synthase and protein tyrosine nitration. Clin Sci.

[36] Anand IS (2006). Relationship between proinflammatory cytokines and anemia in heart failure. European Heart Journal.

[37] Deswal A, Petersen NJ, Feldman AM, Young JB, White BG, Mann DL (2001). Cytokines and cytokine receptors in advanced heart failure: an analysis of the cytokine database from the Vesnarinone trial (VEST). Circulation.

[38] Anand IS, Chandrashekhar Y, Ferrari R, Poole-Wilson PA, Harris PC (1993). Pathogenesis of oedema in chronic severe anaemia: studies of body water and sodium, renal function, haemodynamic variables, and plasma hormones. Heart.

[39] Datta BN, Silver MD (1976). Cardiomegaly in chronic anaemia in rats; gross and histologic features. Indian J Med Res.

[40] Anand I (2004). Anemia and its relationship to clinical outcome in heart failure. Circulation.

[41] Jones DP, Patel J (2018). Therapeutic approaches targeting inflammation in cardiovascular disorders. Biology (Basel).

[42] Frangogiannis NG (2014). The immune system and the remodeling infarcted heart: cell biological insights and therapeutic opportunities. J Cardiovasc Pharmacol.

[43] Heymans S (2009). Inflammation as a therapeutic target in heart failure? A scientific statement from the Translational research committee of the heart failure association of the European Society of cardiology. Eur J Heart Fail.

[44] Katayama T, Nakashima H, Yonekura T, Honda Y, Suzuki S, Yano K (2003). [Significance of acute-phase inflammatory reactants as an indicator of prognosis after acute myocardial infarction: which is the most useful predictor?]. J Cardiol.

[45] Pearson TA (2003). Markers of inflammation and cardiovascular disease: application to clinical and public health practice: A statement for healthcare professionals from the Centers for Disease Control and Prevention and the American Heart Association. Circulation.

[46] Naito Y, Tsujino T, Matsumoto M, Sakoda T, Ohyanagi M, Masuyama T (2009). Adaptive response of the heart to long-term anemia induced by iron deficiency. Am J Physiol Heart Circ Physiol.

[47] Kobak KA (2019). Structural and functional abnormalities in iron-depleted heart. Heart Fail Rev.

[48] Brownlie T, Utermohlen V, Hinton PS, Haas JD (2004). Tissue iron deficiency without anemia impairs adaptation in endurance capacity after aerobic training in previously untrained women123. Am J Clin Nutr.

[49] Maeder MT, Khammy O, Dos Remedios C, Kaye DM (2011). Myocardial and Systemic Iron Depletion in Heart Failure: Implications for Anemia Accompanying Heart Failure. J Am Coll Cardiol.

[50] Xu W, Barrientos T, Mao L, Rockman HA, Sauve AA, Andrews NC (2015). Lethal Cardiomyopathy in Mice Lacking Transferrin Receptor in the Heart. Cell Rep.

[51] O’Meara E (2006). Clinical correlates and consequences of anemia in a broad spectrum of patients with heart failure: results of the Candesartan in Heart Failure: Assessment of Reduction in Mortality and Morbidity (CHARM) Program. Circulation.

[52] Anand IS (2005). Anemia and change in hemoglobin over time related to mortality and morbidity in patients with chronic heart failure: results from ValHeFT. Circulation.

[53] Herzog CA, Muster HA, Li S, Collins AJ (2004). Impact of congestive heart failure, chronic kidney disease, and anemia on survival in the Medicare population. J Card Fail.

[54] McDonagh TA (2021). ESC Guidelines for the diagnosis and treatment of acute and chronic heart failure. Eur Heart J.

[55] Wienbergen H (2016). Usefulness of iron deficiency correction in management of patients with heart failure [from the registry analysis of iron deficiency-heart failure (RAID-HF) registry]. Am J Cardiol.

[56] Belmar Vega L (2016). Investigation of iron deficiency in patients with congestive heart failure: A medical practice that requires greater attention. Nefrologia.

[57] Mistry R, Hosoya H, Kohut A, Ford P (2019). Iron deficiency in heart failure, an underdiagnosed and undertreated condition during hospitalization. Ann. Hematol.

[58] Becher PM (2021). Phenotyping heart failure patients for iron deficiency and use of intravenous iron therapy: data from the Swedish Heart Failure Registry. Eur J Heart Fail.

[59] Lewis GD (2017). Effect of oral iron repletion on exercise capacity in patients with heart failure with reduced ejection fraction and iron deficiency: the IRONOUT HF randomized clinical trial. JAMA.

[60] van Veldhuisen DJ (2017). Effect of ferric carboxymaltose on exercise capacity in patients with chronic heart failure and iron deficiency. Circulation.

[61] Ponikowski P, van Veldhuisen DJ, Comin-Colet J (2015). Beneficial effects of long-term intravenous iron therapy with ferric carboxymaltose in patients with symptomatic heart failure and iron deficiency. Eur Hear J.

[62] Anker SD, Comin Colet J, Filippatos G (2009). Ferric carboxymaltose in patients with heart failure and iron deficiency. N Engl J Med.

[63] Ponikowski P (2014). Beneficial effects of long-term intravenous iron therapy with ferric carboxymaltose in patients with symptomatic heart failure and iron deficiency†. Eur Heart J.

[64] Anker SD (2009). Ferric carboxymaltose in patients with heart failure and iron deficiency. N Engl J Med.

[65] Ponikowski P (2015). Beneficial effects of long-term intravenous iron therapy with ferric carboxymaltose in patients with symptomatic heart failure and iron deficiency†. Eur Heart J.

[66] Ponikowski P (2020). Ferric carboxymaltose for iron deficiency at discharge after acute heart failure: a multicentre, double-blind, randomised, controlled trial. Lancet (London, England).

[67] Anker SD (2018). Effects of ferric carboxymaltose on hospitalisations and mortality rates in iron-deficient heart failure patients: an individual patient data meta-analysis. Eur J Heart Fail.

[68] Ponikowski P (2016). ESC guidelines for the diagnosis and treatment of acute and chronic heart failure: The Task Force for the diagnosis and treatment of acute and chronic heart failure of the European Society of Cardiology (ESC). Developed with the special contribution. Eur J Heart Fail.

[69] Yancy CW (2013). ACCF/AHA guideline for the management of heart failure: a report of the American College of Cardiology Foundation/American Heart Association Task Force on Practice Guidelines. J Am Coll Cardiol.

[70] Brunner-La Rocca H-P, Crijns HJGM (2015). Iron i.v. in heart failure: ready for implementation?. European Heart Journal.

[71] Rizzo C, Carbonara R, Ruggieri R, Passantino A, Scrutinio D (2021). Iron deficiency: A new target for patients with heart failure. Front Cardiovasc Med.

[72] Sindone A (2022). Practical guidance for diagnosing and treating iron deficiency in patients with heart failure: Why, who and how?. J Clin Med.

[73] Bakogiannis C (2020). Iron deficiency as therapeutic target in heart failure: a translational approach. Heart Fail Rev.

[74] Weiss G, Ganz T, Goodnough LT (2019). Anemia of inflammation. Blood.

[75] Anand IS, Gupta P (2018). Anemia and iron deficiency in heart failure. Circulation.

[76] Ponikowski P (2019). Rationale and design of the AFFIRM-AHF trial: a randomised, double-blind, placebo-controlled trial comparing the effect of intravenous ferric carboxymaltose on hospitalisations and mortality in irondeficient patients admitted for acute heart failure. Eur J Heart Fail.

[77] Mentz RJ (2023). Ferric carboxymaltose in heart failure with iron deficiency. N Engl J Med.

[78] ESC (2023). Effects of FCM on recurrent HF hospitalisations: an individual participant data meta-analysis. ESCCongress.

[79] Filippatos EA (2023). Association between hemoglobin levels and efficacy of intravenous ferric carboxymaltose in patients with acute heart failure and iron deficiency: An AFFIRM-AHF subgroup analysis. Circulation.

[80] Filippatos G (2013). Intravenous ferric carboxymaltose in iron-deficient chronic heart failure patients with and without anaemia: a subanalysis of the FAIR-HF trial. Eur J Heart Fail.

[81] Sierpinski R (2021). High soluble transferrin receptor in patients with heart failure: a measure of iron deficiency and a strong predictor of mortality. Eur J Heart Fail.

[82] Grote Beverborg N (2018). Definition of iron deficiency based on the gold standard of bone marrow iron staining in heart failure patients. Circ Heart Fail.

[83] Leszek P (2012). Myocardial iron homeostasis in advanced chronic heart failure patients. Int J Cardiol.

